# Epithelial-mesenchymal crosstalk induces radioresistance in HNSCC cells

**DOI:** 10.18632/oncotarget.23248

**Published:** 2017-12-14

**Authors:** Teresa Bernadette Steinbichler, Abdelmoez Alshaimaa, Metzler Veronika Maria, Dejaco Daniel, Riechelmann Herbert, Dudas Jozsef, Skvortsova Ira-Ida

**Affiliations:** ^1^ Department of Otorhinolaryngology, Medical University of Innsbruck, Innsbruck, Austria; ^2^ Department of Therapeutic Radiology and Oncology, Medical University of Innsbruck, Innsbruck, Austria

**Keywords:** epithelial to mesenchymal transition, chemoresistance, clonogenic assays, ERCC1, survivin

## Abstract

**Objective:**

Epithelial-mesenchymal crosstalk (EMC) contributes to tumor progression, chemoresistance and acquisition of a mesenchymal phenotype (EMT) of cancer cells. This study aims to investigate the effects of EMC on radioresistance in head and neck squamous cell carcinoma (HNSCC) cells.

**Methods:**

In tumor cell lines, the response of HNSCC cells, stimulated with EMC conditioned medium (CM), to irradiation was evaluated with viability and clonogenic assays. Dose modifying factors (DMF) were calculated from the results of clonogenic assays. Potential pathways involved in radioresistance were analyzed with quantitative Real-Time PCR and western blot.

**Results:**

CM significantly reduced the doubling time of SCC-25 cells (from 32.8 hours to 16.8 hours, p=0.0001) and Detroit 562 cells (from 88.5 hours to 29.6 hours, p=0.014). Further it increased clonogenic survival after irradiation. The DMF of CM was 2.04 ± 0.43 (mean ± standard deviation) for SCC-25 cells (p=0.015) and 2.14 ± 0.34 for Detroit 562 cells (p=0.008). Treatment with CM more than tripled the ERCC1 and survivin gene expression in SCC-25 cells.

**Conclusion:**

EMC induced pathways involved in cell survival and DNA repair and led to increased radioresistance in HNSCC cells.

## INTRODUCTION

Radiotherapy is a fundamental treatment modality in head and neck cancer. Local control is achieved by its strong tumoricidal effect. In early stages of laryngeal cancer, irradiation is just as effective as surgery in curing patients [1]. However, failure to radiotherapy is a relevant problem. Radiotherapy failure is mainly due to local recurrence originating from radioresistant tumor cells. For a long time, the causes of irradiation resistance have been primarily searched in intrinsic genotypic or phenotypic characteristics of the cancer cell itself. Cellular mechanisms of radioresistance include for example reduced ability to undergo apoptosis and mutations in DNA repair related genes or alterations of pro-survival signaling pathways. However, there is increasing evidence that crosstalk between cancer cells and the surrounding stroma contributes to epithelial to mesenchymal transition (EMT), chemoresistance, invasion and metastasis [2, 3]. Major components of the tumor stroma are cancer-associated fibroblasts (CAFs), which originate from resident fibroblasts, bone-marrow derived progenitor cells or from cancer cells itself, which underwent EMT. EMT is a reversible cellular process mainly induced by paracrine secretion of small molecules from CAFs [4, 5].

We previously reported that a cell-free, epithelial mesenchymal crosstalk (EMC)-conditioned medium from a tumor cell/fibroblast co-culture could induce EMT and increased Cisplatin resistance in an *in vitro* model of head and neck squamous cell carcinoma (HNSCC) [3]. We further observed that the effect of an EMC-conditioned medium on chemoresistance was not dependent on the acquisition of a mesenchymal phenotype (EMT). We hypothesized that chemoresistance and EMT are two different effects induced by EMC [3].

In this study, we investigated whether EMC induces irradiation resistance in HNSCC cells in a similar setup using SCC-25 and Detroit 562 cells. SCC-25 cells were originally isolated from the primary tumor of a patient with tongue carcinoma [6, 7]. SCC-25 cells form tumors in severe combined immunodeficiency (SCID) mice but not in athymic nude mice suggesting less aggressive behavior. Otherwise, xenografted SCC-25 cells do not develop regional or distant metastases in mouse models [8]. In contrast, Detroit 562 cells grow tumors and develop regional and lung metastases when injected in nude mice [9]. Detroit 562 was isolated from the malignant pleural effusion of a patient with pharyngeal carcinoma who was treated with radiochemotherapy prior to metastasis, which means that an already radioresistant phenotype was collected [10, 11]. We stimulated these cell lines with cell-free EMC conditioned medium from a mix-culture of tumor cells and fibroblasts (CM). The response to irradiation was assessed after exposure to increasing irradiation doses with viability and clonogenic assays.

## RESULTS

### EMC conditioned medium (CM) reduced the doubling time of HNSCC cells

**Figure 1:**
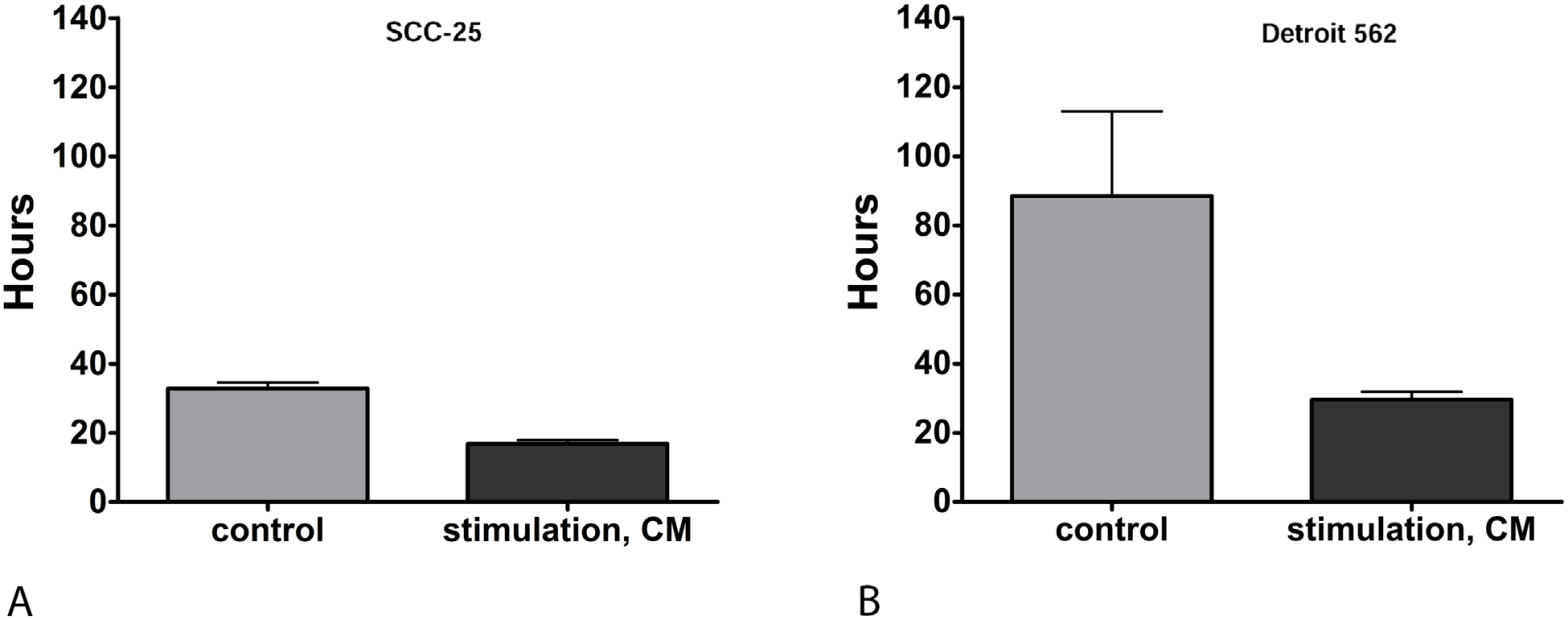
**(A)** Doubling time of SCC-25 in hours: Doubling times were calculated in non-irradiated cells. Control: following treatment of SCC-25 cells with standard albumin medium. CM: after treatment of SCC-25 with co-culture conditioned medium. Stimulation with CM reduced the doubling time in SCC-25 cells from 32.8 +/- 2.4 hours to 16.8 +/- 1.6 hours compared to the control medium (p=0.0001). **(B)** Doubling time of Detroit 562 in hours: Control: after treatment of Detroit 562 cells with standard albumin medium. CM: after treatment of Detroit 562 with co-culture conditioned medium. In Detroit 562 cells, stimulation with CM reduced doubling time from 88.5 +/- 34.7 hours (mean +/- SD) to 29.6 +/- 3.3 hours compared to the control medium (p= 0.014).

SCC-25 and Detroit 562 cells were stimulated with CM or control medium for three days as described below. Doubling time of cells was calculated from the results of viability assays of irradiation controls receiving 0 Gy. Stimulation with CM significantly reduced doubling time in both cell lines, which means that this treatment increased cell proliferation. Stimulation with CM reduced the doubling time in SCC-25 cells from 32.8 ± 2.4 hours (control; mean ± SD) to 16.8 ± 1.6 hours (CM, p=0.0001; Figure 1A). In Detroit 562 cells, stimulation with CM reduced doubling time from 88.5 ± 34.7 hours (control) to 29.6 ± 3.3 hours (CM; p= 0.014; Figure 1B).

### EMC conditioned medium (CM) contained high concentrations of IL-6 and IL-6 increased cell proliferation

CM contained high concentrations of IL-6 (1.340 ng/ml, data not shown). A pure cancer cell culture was stimulated with IL-6 (50 ng/ml) according to Sullivan et al [12]. IL-6 stimulation increased cell viability in MTT assays from 1.18 ± 0.12 to 1.95 ± 0.16 compared with controls in SCC 25 cells (p<0.0001). In Detroit 562 cells IL-6 stimulation increased cell viability from 1.92 ± 0.12 to 2.15 ± 0.18 (p=0.001). CM increased, in the same experimental setting, cell viability in SCC-25 cells to 1.32 ± 0.2 (p<0.01) and in Detroit 562 cells to 2.17 ± 0.06 (p<0.0001) compared to control cells. There was no statistical difference in the viability increase due to stimulation with CM and IL-6 in Detroit 562 cells (p=0.7). In SCC-25 cells, IL-6 stimulation increased cell viability to a greater extent than CM (p<0.0001).

### EMC conditioned medium (CM) induced epithelial to mesenchymal transition - like gene expression pattern and increased gene expression of ERCC1 and survivin in SCC-25 cells

**Figure 2:**
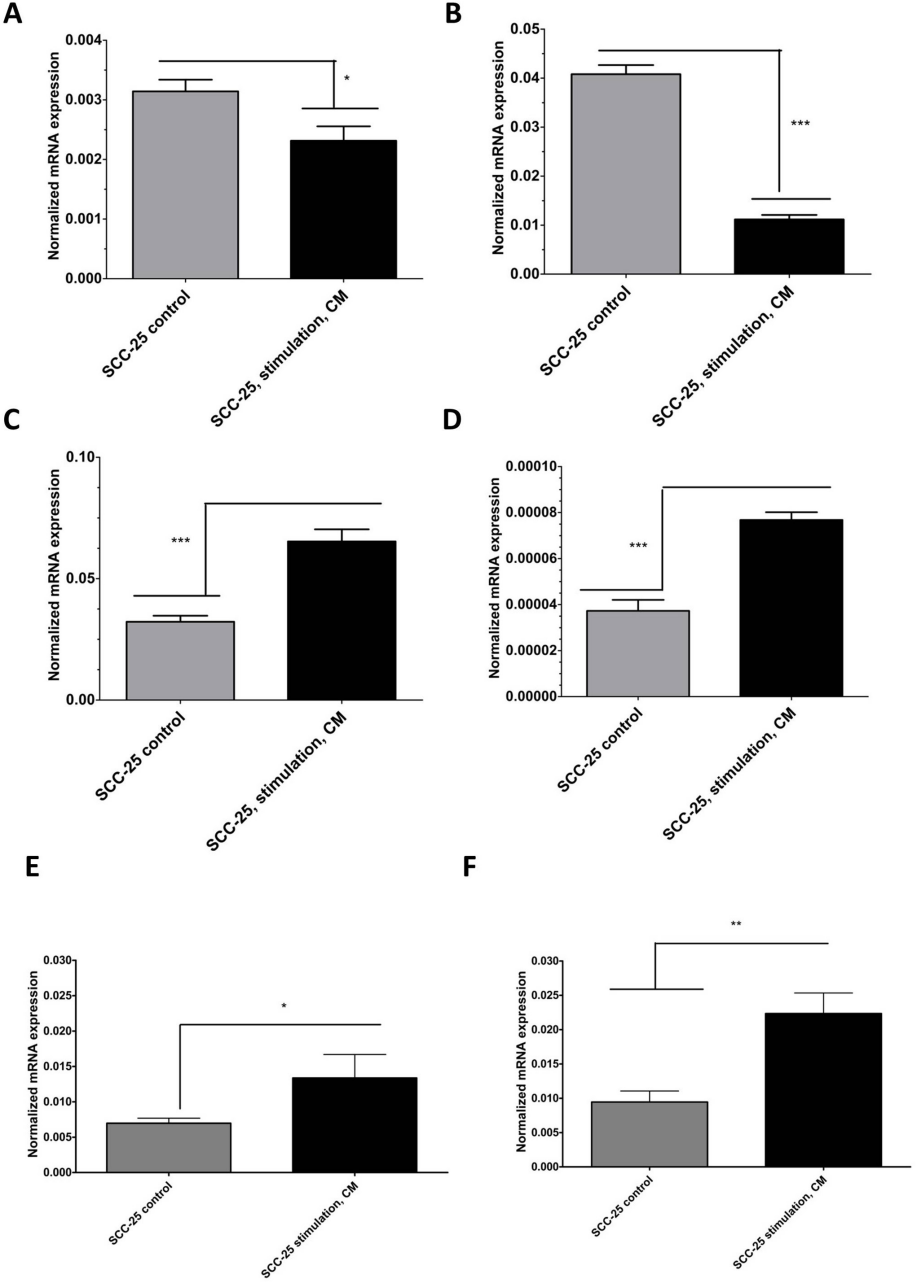
Real-time PCR analysis of EMT-like (**A-D**) and radioresistance - related (**E-F**) gene expression changes in SCC-25 cells in control and CM-treated conditions. SCC-25 cells were treated for 72 hours with CM, followed by total RNA isolation, reverse transcription, and real-time PCR. Relative gene expression was calculated with the −δδCt method for E-cadherin (A), desmoplakin (B), vimentin (C), MMP-9 (D), ERCC1 (E), and survivin (F) in control and CM-treated conditions, where β-actin was used as housekeeping gene.

As reported previously, stimulation with CM induced EMT-like gene expression changes in SCC-25 cells [3]. Stimulation with CM reduced the relative mRNA expression of the epithelial differentiation markers E-cadherin about 85% and desmoplakin about 78% (Figure 2A–2B; p < 0.05) Relative mRNA expression of mesenchymal genes as vimentin and matrix metalloproteinase-9 (MMP-9) [13], increased six fold after stimulation with CM (Figure 2C–2D; p < 0.05). CM stimulation did not induce significant changes in EMT-like gene expression patterns in Detroit 562 cells (data not shown). To elucidate the mechanistic background of CM-induced radioresistance, we hypothesized that CM might influence the expression of genes involved in DNA repair or genes related to cell survival. Excision repair cross complementation group 1 (ERCC1) is a DNA-damage repair associated gene, which is involved in resolution of irradiation induced DNA breaks [14]. In SCC-25 cells, stimulation with CM increased the relative ERCC1 gene expression 5-fold (p < 0.01; Figure 2E). In contrast, there was no increase in ERCC1 gene expression of Detroit 562 cells after CM stimulation (p=0.065). The base line gene expression of ERCC1 in Detroit 562 cells was slightly higher than in SCC-25 cells but it was not significantly different (p=0.16).

Additionally, the potential of CM to induce upregulation of an anti-apoptotic gene was assessed. Anti-apoptotic gene upregulation might support G2-phase arrest, allow repair functions, and inhibit apoptosis [15]. Survivin is such an anti-apoptotic gene and its relative mRNA expression increased three fold in SCC-25 cells after stimulation with CM (p < 0.05; Figure 2F), whereas there was no increase in survivin gene expression of Detroit 562 cells after CM stimulation (not shown). The basic survivin gene expression was 2 times higher in Detroit 562 cells than in SCC-25 cells (p < 0.001). These results indicate that CM might support genes involved in DNA-repair or in improved cell survival.

**Figure 3:**
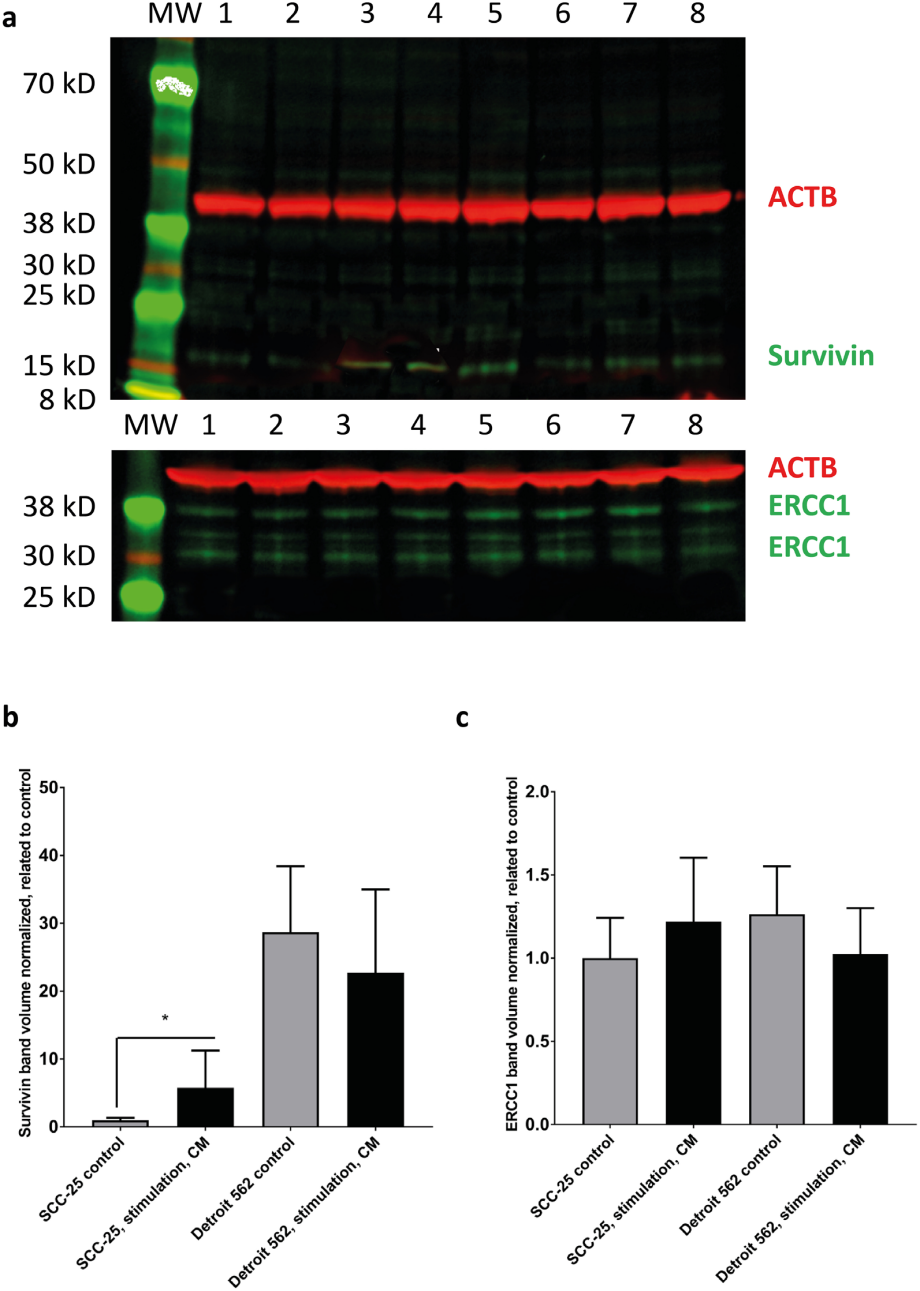
Western blot analysis of survivin and ERCC1 in SCC-25 and Detroit 562 cells in control and CM - treated conditions SCC-25 and Detroit 562 cells were treated with albumin - containing (control) or with CM medium (stimulation, CM) and subjected to western blot analysis of β-actin (red), survivin (green) or ERCC1 (green) in a two channel near infrared fluorescence detection system. β-actin loading control (42 kD), a single band was detected at 700 nm and is presented in red, survivin or ERCC1 were detected at 800 nm and are presented in green. (**a**) Survivin was detected as a single band in both SCC-25 (lanes 1-4) and Detroit 562 cells (lanes 5-8). ERCC1 was detected as multiple bands with two more intensive ones at 38 and 30 kD in both cell lines. More bands might be present in certain cell lines, which was also described by the data sheet of the antibody provider. Lanes 1-2: SCC-25 control, lanes 3-4: SCC-25 stimulation with CM, lanes 5-6: Detroit 562 control, lanes 7-8: Detroit 562 stimulation with CM; MW: molecular weight standard. (b-c) Column diagrams representing mean and standard error of measurement of normalized (to loading control, β-actin) survivin (**b**) and ERCC1 (**c**) band volumes from 8 gel lanes for each SCC-25 and Detroit 562 control and CM stimulated protein samples. The mean band volume of SCC-25 control was set as “1”. ^*^: p < 0.05.

Using near infrared fluorescent antibody detection and chemiluminescence, the changes in mRNA expression were also tested at protein level with western blot. Alterations in E - cadherin protein level were too low to be detected in protein level with near infrared fluorescent antibody detection and Vimentin expression was weak with this method (Figure 3). Western blots performed with conventional horseradish peroxidase chemiluminescence demonstrated an increased 46 kD vimentin band after CM treatment. E-cadherin showed a marginal decrease after treatment with CM in SCC-25 cells. In Detroit 562 cells CM did not induce changes in vimentin expression (46 kD band). E-cadherin showed a marginal decrease after treatment with CM (3).

The 15 kD survivin specific band showed a significant increase in SCC-25 cells after stimulation with CM (Figure 3a–3b), when normalized to β-actin loading control. In Detroit 562 cells there was a higher constitutive detection level of 15 kD survivin, but no differential regulation was observed after stimulation with CM (Figure 3a–3b). ERCC1 was at the detection limit of the antibody in both cell lines (Figure 3a, 3c). There was an increase after stimulation with CM, but it was not significant (Figure 3c).

### EMC conditioned medium (CM) did not increase cell viability following irradiation

Cell viability assays are not the standard tool to identify tumoricidal effects of radiotherapy [16]. Viability fraction decreased in both cell lines with higher irradiation dose and longer time interval. This decrease was not significantly altered in CM stimulated SCC-25 and Detroit 562 cells (data not shown). Following stimulation of SCC-25 and Detroit 562 cells with CM or control medium, cells were irradiated with increasing doses from 0 Gy (control irradiation) to 10 Gy. Cell viability was measured after 24, 48, and 72 hours.

### EMC conditioned medium (CM) increased clonogenicity after irradiation

**Figure 4:**
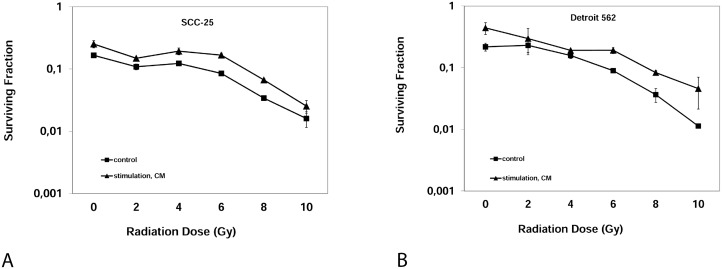
Clonogenic Assay: Surviving fraction of SCC-25 cells (**A**) or Detroit 562 cells (**B**) after irradiation with 0, 2, 4, 6, 8, 10 Gray after three weeks. Control: Albumin medium treated, stimulation, CM: EMC-conditioned medium.

To analyze the effect of CM on radioresistance of HNSCC cells, clonogenic assays were performed. Clonogenic assay measure reproductive integrity of cancer cells but not cell proliferation and are considered the standard measurement tool for evaluating tumoricidal effects of radiotherapy *in vitro* [16]. Clonogenicity, displayed as surviving fraction, was significantly increased after stimulation with CM compared to stimulation with control medium for each irradiation dose from 2-10 Gy in both cell lines (Figure 4). The surviving fraction following 6 Gy increased after stimulation with CM from 8.4 ± 0.6% to 16.7 ± 1.3% in SCC-25 cells (p<0.001). Analogously, the surviving fraction after 6 Gy increased after the stimulation with CM from 8.9 ± 1.1% to 19.1 ± 3.8% in Detroit 562 cells (p=0.01). From clonogenic assays, dose-modifying factors (DMF) as a measure of radioprotection were calculated as described by Rosenberg [16]. The DMF for CM differed from a DMF of 1 (no effect) in both cell lines: The DMF was 2.04 ± 0.43 (mean ± SD, p=0.015) in SCC-25 cells; the DMF for CM was 2.14 ± 0.34 in Detroit 562 cells (p=0.008).

## DISCUSSION

Epithelial-mesenchymal crosstalk (EMC), i.e. the communication between cancer cells and the surrounding stroma, relies on paracrine signaling, cell-cell interactions and cell-matrix interactions. Soluble products of EMC may be transferred by an EMC conditioned medium to cancer cell cultures, where they exert EMC related effects including increased cancer cell mobility [17], invasiveness [17], acquisition of a mesenchymal phenotype (EMT) [3], generation of cells with stem-like properties [18] and chemoresistance [3]. EMC related effects may be associated with EMT, but also occur without EMT [19]. This is supported by several experiments: for instance, we observed EMC related cisplatin resistance in association with EMT in SCC-25 cells, but as an EMT independent effect in Detroit 562 cells. Moreover, TGF-ß1 (transforming growth factor-ß1) induced EMT but not cisplatin resistance in SCC-25 cells [3]. TGF-ß1 is found in high concentrations in CM (106 ± 5.82 pg/ml, data not shown). Accordingly, TGF-ß1 induced migration in ovarian cancer cells independent of acquisition of a mesenchymal phenotype [19]. EMC is thought to be a relevant process leading to cancer progression and treatment resistance, but these effects do not necessarily depend on the acquisition of a mesenchymal phenotype of cancer cells [20]. Therefore, we consider EMT an insufficiently comprehensive term for EMC related effects in general.

### EMC induced radioresistance in two HNSCC cell lines

The task of this study was to analyze if EMC increases radioresistance in two HNSCC cell lines. We found that EMC had no influence on cell viability in the first 72 hours after irradiation but affected radioresistance in clonogenic assays. Viability assays measure acute toxic effects of irradiation. More important from a clinical point of view are long-term effects of irradiation on cancer cell survival, e.g. growth inhibition due to cell cycle arrest and subsequent apoptosis, without affecting the viable cell number in the short term. These long-term effects prohibit rapid repopulation and locoregional recurrence. They are best measured with clonogenic assays [21]. They assess if tumor cell maintain their reproductive integrity [16]. Consequently, clonogenic assays are considered the reference method for assessing radiosensitivity *in vitro* [22]. EMC increased clonogenicity following irradiation in both cell lines, resulting in a DMF of 2.04 ± 0.43 (mean ± SD; p=0.015) for SCC-25 cells and a DMF of 2.14 ± 0.34 (p=0.008) in Detroit 562 cells, suggesting that EMC increased radioresistance in HNSCC *in vitro*. A DMF of 1 or more is considered biologically relevant. Skvortsova and co-authors induced radiation-resistant HNSCC cells from parental HNSCC cells by repeated exposure to ionising radiation (10 Gy). These cells received this treatment 10 times every 2-3 weeks resulting in a total dose of 100 Gy. The HNSCC cell clones recovering after exposure to this total dose were considered radioresistant. These radioresistant HNSCC cells were again irradiated and compared with control HNSCC cells. The DMF of the radioresistant cells and the parental HNSCC cells varied between 1.4 and 2 [23].

Our results are in line with recent studies, which revealed a significant role of tumor microenvironment in the development of resistance to radiotherapy: De Jong and colleagues analyzed micro RNA and mRNA expression in a large panel of HNSCC cells. They found that EMT associated gene expression caused intrinsic radioresistance of HNSCC cells by prolonging the time spent in G2-phase, leading to a more efficient DNA double strand break repair and autophagy as a mechanism to evade cell death [24]. Similar results were obtained by Chang et al., who found that an increased EMT activation and acquisition of stem cell like features led to radioresistance in prostate cancer cells via activation of the PI3K/Akt/mTOR signaling pathway [25].

All these effects were EMT dependent. Here we describe that an EMC conditioned-medium was able to induce radioresistance in HNSCC cells *in vitro*. This effect was not EMT dependent, but a result of EMC. Radioresistance of Detroit 562 cells was increased by CM despite the lack of induction of a mesenchymal phenotype. A possible mediating factor is TGF-β1, which is able to induce radioresistance in an EMT-independent manner [19, 20].

CM stimulated cell proliferation, which is considered to make cells more sensitive for irradiation according to the law of Bergonié and Tribondeau [26–28]. The observation of increased radioresistance could therefore be biased by the fact that CM treated proliferating cells acquire full confluence more quickly, which in turn may cause increased radioresistance [29]. To rule out that increased resistance is due to confluent or overloaded culture dishes, we set the seeded cell number low enough that confluence was not even reached in control cells.

### EMC activated DNA-repair and anti-apoptotic pathways

Treatment with CM induced a 4.5-fold increase of ERCC1 (p=0.03) in SCC-25 cells. ERCC1 is involved in the repair of DNA-damage caused by ionizing radiation through nucleotide excision repair (NER). ERCC1-deficient cells are more sensitive to double strand breaks (DSB) produced by irradiation even if NER is not directly involved in the repair of DNA-DSB. Vice versa, overexpression of ERCC1 could lead to radioresistance [30]. The increased expression of ERCC1 could explain the increased radioresistance caused by CM-stimulation in SCC-25 cells.

Furthermore, stimulation with CM increased survivin expression in SCC-25 cells 3.2- fold (p<0.01). Survivin is known as a multi-functional protein, which participates in at least three homeostatic networks including mitosis regulation, apoptosis inhibition [31, 32] and cellular stress response [33]. The association between survivin expression and radiosensitivity has been described in lung [34], sarcoma [35] and non-small cell lung [36] cancer cell lines. Survivin inhibits apoptosis following irradiation and consequently causes cancer cell survival [37]. The increased expression of survivin, following CM stimulation could be another explanation for the increased radioresistance of SCC-25 following stimulation with CM.

In Detroit 562 cells no effect of CM stimulation on ERCC1 and survivin expression was observed. Detroit 562 cells were isolated from radioresistant metastatic cells [10, 11] and genes involved in radioresistance could already be upregulated in this cell line. Baseline ERCC-1 and survivin expression were higher in Detroit 562 than in SCC-25 cells. Furthermore, despite having already collected a radioresistant phenotype when collecting Detroit 562 cells, CM was still able to increase radioresistance. It is possible that CM induces the expression of other anti-apoptotic signaling pathways, which were not analysed in our experimental setting but are of relevance in EMC-mediated radioresistance in Detroit 562 cells. One example could be increased IL-6 expression after stimulation with CM. CM contains high levels of IL-6, which promotes DNA-repair and prevents apoptosis after irradiation in non-small lung cancer (NSCLC)-cells by affecting the self-renewal capacity of cancer stem cells [38].

### EMC increased proliferation of HNSCC cells

CM significantly reduced doubling times of non-irradiated HNSCC cells *in vitro*, which indicates that CM increased cell proliferation. CM halved the doubling time of SCC-25 cells (p=0.0001), i.e. it increased proliferation about two-fold. In Detroit 562 cells, CM reduced the doubling time to 1/3 (p=0.014). This effect is likely due to humoral factors released from CAFs contained in the co-culture, from which the CM derived, which means it is an effect of EMC. Interleukin-6 (IL-6) in EMC is a likely signaling molecule mediating this effect. Stimulation of SCC-25 and Detroit 562 with IL-6, in similar concentrations as measured in CM, was able to induce a significant increase in cell viability in both cell lines (p<0.001). In Detroit 562 cells the effect of IL-6 on cell viability was comparable to that of CM. In SCC-25 cells, IL-6 had a greater effect on cell viability increase than CM (p<0.0001).

CAFs promote cancer cell proliferation in various cancer types including breast cancer, and head and neck cancer [3, 39]. In contrast, Gonçalves-Ribeiro and coworkers observed an antiproliferative effect of CAF-conditioned medium on colorectal carcinoma and hepatic carcinoma cells as a result of a prolonged G1-phase transit. This anti-proliferative effect of conditioned medium on cancer cells resulted in enhanced resistance to chemotherapeutic agents such as oxaliplatin and 5-FU [40, 41]. Various factors including different tumor cell responsiveness and heterogenic CAF populations in the different cancer types may explain these differences. Notably, we observed CM induced platinum resistance in HNSCC cells despite increased proliferation [3].

### EMC induced EMT in SCC-25 cells

CM upregulated vimentin and MMP-9 gene expression, and downregulated E-cadherin and desmoplakin gene expression in SCC-25 cells. As expected for an epithelial cell line, baseline vimentin and MMP-9 expression was low and baseline E-cadherin and desmoplakin expression was high. Stimulation with CM reduced the mRNA expression of the epithelial differentiation marker E-cadherin by about 85% (Figure 2A; p < 0.05) and desmoplakin by about 77% (Figure 2B; p < 0.05). Mesenchymal gene mRNA expression of vimentin and MMP-9 increased six fold after stimulation with CM (Figure 2C–2D; p < 0.05).

The fact that EMT can be induced by cell free medium supports the concept that EMT mainly depends on paracrine signalling [42–44]. Beyond upregulation of mesenchymal and downregulation of epithelial markers, CM increased cell viability in SCC-25 cells (p<0.01) and Detroit 562 cells. EMT-associated increase of cell viability is in line with several recent studies [5, 45].

CM failed to induce EMT-like phenotypic changes in Detroit 562 cells. Despite a lack of phenotypic changes, CM increased the cell viability of Detroit 562 cells (p=0.001), supporting the concept that EMT-phenotype induction and changes in cell viability may be caused by different factors but both are a consequence of EMC.

As described before TGF-β1 might be a factor responsible for acquiring a mesenchymal phenotype, but had no or a negative influence on cell viability [3]. IL-6 increased cell viability in our experimental setting, but is not known to be involved in the acquisition of a mesenchymal phenotype. IL-6 activates proinflammatory-signaling pathways in epithelial cancers and leads to cancer progression, metastasis and therapy resistance through activation of pro-survival signals [46–49]. The effects of CM and IL-6 on cell viability imply that observed changes in cell viability are not caused by the acquisition of a mesenchymal phenotype (EMT), but rather an additional effect of EMC in the tumor microenvironment.

## MATERIALS AND METHODS

### Cell lines

Human gingival fibroblasts (HGF) were purchased from Cell Line Service, Eppelheim, Germany [6, 50]. They were cultured in DMEM-low glucose supplemented with 10% fetal bovine serum (FBS, both PAA, Pasching, Austria), 2 mM l-glutamine, 100 units/ml penicillin, and 100 μg/ml streptomycin. SCC-25 were purchased from German Collection of Microorganisms (DSMZ, Braunschweig, Germany) and Detroit 562 cells from the Cell Lines Service (Eppelheim, Germany), and were cultured in DMEM/F12 supplemented with 10% FBS, 2 mM l-glutamine (both PAA, Pasching, Austria), 100 units/ml penicillin and 100 μg/ml streptomycin [6]. All cell lines were cultured at 36.9 °C under 5% CO_2_ and 96% humidity.

### EMC conditioned medium (CM)

To produce CM, 4 x 10^4^/ml SCC-25 or Detroit 562 cells and 1 x 10^4^ HGF cells/ml were plated in 250 ml cell culture flasks and cultured for 72 hours in 15 ml fetal bovine serum-containing medium (1:1 mix of DMEM/F12 and DMEM-low glucose supplemented with 10% fetal bovine serum (FBS), 2 mM l-glutamine, 100 units/ml penicillin and 100 μg/ml streptomycin). Then the cells were washed twice with Dulbecco’s Phosphate- Buffered Saline (DPBS; Biowhittaker®, Belgium) and the serum-containing medium was replaced by 15 ml albumin-containing medium (7,5 ml DMEM/F12 and 7,5 ml DMEM-low glucose supplemented with bovine serum albumin (BSA); 0.4 g albumin/100 ml medium) replacing the protein content of 10% FBS, 2 mM l-glutamine, 100 units/ml penicillin and 100 μg/ml streptomycin. Albumin-containing medium was left for 48 hours on the co-culture allowing interacting epithelial cells and fibroblasts to secrete EMC-related factors into the medium. Afterwards, CM was collected and cells were counted. CM was portioned per cell number as described by Hassona et al. [44]. CM was sterile-filtered and stored at −20°C. The composition of the conditioned medium was estimated semiquantitatively using a human cytokine antibody array C6 following the instructions of the manufacturer (Raybiotech, Inc., Norcross, GA, USA).

### Stimulation of SCC-25/Detroit 562 cells with EMC conditioned medium

To induce EMC, SCC-25 and Detroit 562 cells were treated with 7 ml CM per 50 ml cell culture flask for 72 hours. The medium was changed daily. As control stimulation, cells were treated with a control medium (albumin medium) and identical medium changes. At the end of the stimulation period, the stimulated cells were used for RNA extraction, protein isolation, viability assays and clonogenic assays.

### RNA isolation, reverse transcription and real time quantitative PCR (qPCR)

For RNA isolation, cells were collected, lysed in TRIzol® Reagent (Ambion®, Life technologiesTM, Carlsbad, California, USA) and RNA was isolated as instructed by the manufacturer of TRIzol. RNA concentrations were determined by photometric measurements (BioPhotometer plus 6132, Eppendorf, Germany). The total RNA was reverse transcribed by M-MuLV Reverse Transcriptase (GeneON, Ludwigshafen am Rhein, Germany) in a MyiQ™ cycler (BIO-RAD Laboratories, Inc., USA) following the manufacturer’s instructions. Real time quantitative PCR (qPCR) of cDNA transcripts was performed in a MyiQ™ cycler using iTaq™ Universal SYBR™ Green Supermix (BIO-RAD Laboratories, Inc., USA). Primers were synthesized by Invitrogen™ (Darmstadt, Germany). Specificity of PCR products was confirmed by agarose gel electrophoresis (single band product) and PCR products were sent for Sanger sequencing to service provider (Microsynth, Vienna, Austria). Beta - glucuronidase (*GUSB*) showed comparable Ct-values to the genes of interest and was used as a reference gene in relative real-time qPCR-based quantification of mRNA expression. To exclude that GUSB might have been regulated, we tested the GUSB mRNA expression against β-actin in all treatment conditions in all cells, and it was found not significantly different in any condition (p > 0.05) [51]. The relative gene expression changes after CM treatment compared to controls were similar when GUSB or β-actin were used as reference. Each experiment was performed with twelve independent replicates.

### Protein isolation and western blotting

Following treatments SCC-25 and Detroit 562 cells were washed twice with DPBS and scraped into 100 μl RIPA-buffer (50 mM Tris HCl/pH:8.0, 2 mM EDTA, 1 mM EGTA, 1% Triton X-100, 0.25% sodium deoxycholate, 0.1% sodium dodecylsulfate, 150 mM NaCl, 10 mM NaF, 1 mM PMSF)/10^6^ cells (all from Sigma, Vienna Austria). The cell suspension was vortexed and incubated 3-times for 15 minutes on ice, homogenized in 22G needles and centrifuged at 15000 g, 15 minutes, 4 °C. The cleared supernatant was subjected to protein concentration measurement using the Pierce 660 nm protein assay (Pierce, Rochford, IL, USA) according to the instructions of the manufacturer. 20 μg protein from all samples was subsequently processed for western blot analysis using ready Nupage 4-12% gradient gels (Life Technologies, Carlsbad, CA, USA), and Bolt electrophoresis and western blot apparatus (Life Technologies) following the instructions of the manufacturer. Blocking (one hour at room temperature) and primary antibodies (overnight at 4°C) were applied in Starting Block (TBS) buffer (Life Technlogies) using primary rabbit monoclonal anti-survivin (Abcam, Cambridge UK) at 1: 2500 or rabbit monoclonal anti-ERCC1 (Cell Signaling Technology, Danvers, MA, USA, Cat. Nr. 12345) at 1: 1000, combined with mouse IgM raised against β-actin (Proteintech, Manchester, UK) at 1: 15000. After 5 washes in TBS containing 0.02% Tween 20, signal was detected with anti-rabbit-IgG-IRDye-800CW (1: 15000) and anti-mouse-IgM-IRDye 680 RD (1: 25000) (Li-cor, Bad Homburg, Germany) after one hour incubation at room temperature, and 5 washes in in TBS containing 0.02% Tween 20. The near infrared fluorescence signal was imaged by an Azure C500 documentation system (Biomedica, Vienna, Austria), quantification was performed using Li-cor Image Studio Lite 5.2, band volumes of protein of interest and β-actin were collected and the protein of interest was normalized to β-actin. Each experiment was performed in two replicates. In each experiment the normalized volume of the proteins of interest in SCC-25 control samples was set at “1”. This enabled a comparison between controls and treatments and between Detroit 562 and SCC-25 cells. The proteins of interest were not detected in less than 20 μg total protein extract.

### Irradiation of cells

Following stimulation (CM or control medium), SCC-25 and Detroit 562 cells were treated with increasing irradiation doses of 0 Gy (control), 2 Gy, 4 Gy, 6 Gy, 8 Gy, and 10 Gy in one fraction with a dose rate of 2.7 Gy per minute. A 6 MV photon beam from a Varian Clinac 2100 linear accelerator (Varian Medical Systems, Inc., Palo Alto, California, USA) was used. For clonogenic assays, 2 × 10^4^ cells were plated in 250 ml cell culture flasks. After stimulation, cells were treated with 0 Gy, 2 Gy, 4 Gy, 6 Gy, 8 Gy or 10 Gy with the same treatment modalities. Cell culture media were changed daily.

### Viability assay

SCC-25 and Detroit 562 were plated at concentrations of 60 000 cells per well. Cells were either stimulated with CM or control medium as described above. Following irradiation, cells were incubated for 24 h, 48 h or 72 h at 37°C and then trypsinized and counted using a Beckman Coulter Vi-CELL AS cell viability analyzer (Beckman Coulter, Fullerton, CA). The doubling time (DT) for each cell line was determined using the following equation:

$$\text{DT}\left(\text{hours}\right)=0.693\left(\text{t}-{\text{t}}_{0}\right)/\ln\left(\text{Nt}/{\text{N}}_{0}\right),$$

where t_0_ is the time at which exponential growth began, t time in hours, Nt the cell number at time t, and N_0_ the initial cell number (53). Cell viability was expressed as viability fraction of irradiated cells relative to control cells treated with 0 Gy and stimulated either with CM or control medium. Each experiment was repeated in three independent sets with at least six biological repeats.

### MTT- assay

Cell viability was evaluated by MTT-assays using the tetrazolium salt method. The MTT-assay (Roche, Vienna) is a quantitative colorimetric method used to determine metabolic activity [53]. After three days of IL-6 treatment (50 ng/ml, R&D Systems, Minneapolis, USA.), 10 μl of 5 mg/ml MTT salt (in DMEM/F12 supplemented with 10% FBS, 2 mM l-glutamine, 100 units/ml penicillin, and 100 μg/ml streptomycin) was administered to the cells (100 μl). Alternatively, cells were treated with control medium as described above. Cells were incubated for 4 h at 37°C, followed by the dissolution of the formazan reaction product using 10% sodium dodecylsulphate in 10 mM HCl at 37°C for 12 hours. Absorbance at 550 nm was measured with a microtiter plate reader (Athos 2010, Salzburg, Austria). The MTT-tests were performed in four independent sets containing at least six biological repeats.

### Clonogenic assay

For analysis of the anti-clonogenic effect of irradiation, we used a modified clonogenic assay described by Puk and coworkers [22]. SCC-25 and Detroit 562 cells were washed with PBS and cultured in 250 ml tissue culture flasks in DMEM/F12 supplemented with 10% FBS, 2 mM l-glutamine, 100 units/ml penicillin, and 100 μg/ml streptomycin at a concentration of 1000 cells/flask for 21 days. After 21 days, the cultures were fixed and stained in 0.5% gentian violet (Sigma-Aldrich, Darmstadt; Germany), dissolved in methanol (Carl Roth; Karlsruhe, Germany) and colonies with more than 50 cells were counted with a ColCount colony counter (Oxford Optronix, Oxford, United Kingdom). The surviving fraction (SF) was calculated by the following formula:

SF=number of colonies formed/(number of cells seeded×plating efficiency of the control group),

where the plating efficiency was calculated as the ratio between colonies observed and number of cells plated [16].

Dose–response clonogenic survival curves were plotted on a log-linear scale. To quantify the effect of CM stimulation on cancer cell radioresistance, data from the surviving fraction curve were used to calculate the dose-modifying factor (DMF) [16]. DMFs were calculated as the dose to reach the iso-survival of 10% in CM-stimulated cells divided by the dose to reach the same survival in the control cells [16, 52, 54]. The clonogenic assays were performed in three independent replicates with at least six replicates.

### Data analysis

Data were presented as mean +/- standard deviation (SD) unless indicated otherwise. The results of real time PCR analysis were analyzed with GraphPad Prism 4.03 (GraphPad Software Inc, San Diego, CA, USA). Mean values among groups were compared with unpaired *t*-tests or with Mann-Whitney tests if indicated. Viability changes in CM stimulated cells *vs*. controls were tested with unpaired *t*-test. For evaluation of clonogenic assays, DMFs were calculated as described above and tested with one-sample t-tests. SPSS-22 was used (IBM Corporation, Armonk, NY, USA) for the data analysis.

## References

[1] Warner L, Chudasama J, Kelly CG, Loughran S, McKenzie K, Wight R, Dey P (2014). Radiotherapy versus open surgery versus endolaryngeal surgery (with or without laser) for early laryngeal squamous cell cancer. Cochrane Database Syst Rev.

[2] Skvortsova I, Debbage P, Kumar V, Skvortsov S (2015). Radiation resistance: cancer stem cells (CSCs) and their enigmatic pro-survival signaling. Semin Cancer Biol.

[3] Steinbichler TB, Metzler V, Pritz C, Riechelmann H, Dudas J (2015). Tumor-associated fibroblast-conditioned medium induces CDDP resistance in HNSCC cells. Oncotarget.

[4] Smith A, Teknos TN, Pan Q (2013). Epithelial to mesenchymal transition in head and neck squamous cell carcinoma. Oral Oncol.

[5] Orimo A, Weinberg RA (2006). Stromal fibroblasts in cancer: a novel tumor-promoting cell type. Cell Cycle.

[6] Dudas J, Bitsche M, Schartinger V, Falkeis C, Sprinzl GM, Riechelmann H (2011). Fibroblasts produce brain-derived neurotrophic factor and induce mesenchymal transition of oral tumor cells. Oral Oncol.

[7] Rheinwald JG, Beckett MA (1981). Tumorigenic keratinocyte lines requiring anchorage and fibroblast support cultured from human squamous cell carcinomas. Cancer Res.

[8] Chen YW, Lin GJ, Chia WT, Lin CK, Chuang YP, Sytwu HK (2009). Triptolide exerts anti-tumor effect on oral cancer and KB cells in vitro and in vivo. Oral Oncol.

[9] Sano D, Xie TX, Ow TJ, Zhao M, Pickering CR, Zhou G, Sandulache VC, Wheeler DA, Gibbs RA, Caulin C, Myers JN (2011). Disruptive TP53 mutation is associated with aggressive disease characteristics in an orthotopic murine model of oral tongue cancer. Clin Cancer Res.

[10] Peterson WD, Stulberg CS, Simpson WF (1971). A permanent heteroploid human cell line with type B glucose-6-phosphate dehydrogenase. Proc Soc Exp Biol Med.

[11] Peterson WD, Stulberg CS, Swanborg NK, Robinson AR (1968). Glucose-6-phosphate dehydrogenase isoenzymes in human cell cultures determined by sucrose-agar gel and cellulose acetate zymograms. Proc Soc Exp Biol Med.

[12] Sullivan NJ, Sasser AK, Axel AE, Vesuna F, Raman V, Ramirez N, Oberyszyn TM, Hall BM (2009). Interleukin-6 induces an epithelial-mesenchymal transition phenotype in human breast cancer cells. Oncogene.

[13] Tobar N, Villar V, Santibanez JF (2010). ROS-NFkappaB mediates TGF-beta1-induced expression of urokinase-type plasminogen activator, matrix metalloproteinase-9 and cell invasion. Mol Cell Biochem.

[14] Ciaparrone M, Caspiani O, Bicciolo G, Signorelli D, Simonelli I, de Campora L, Mazzarella G, Mecozzi A, Pianelli C, Camaioni A, Catalano P, Pasqualetti P, Fabiano A (2015). Predictive role of ERCC1 expression in head and neck squamous cell carcinoma patients treated with surgery and adjuvant cisplatin-based chemoradiation. Oncology.

[15] Hu J, Pan J, Luo Z, Tao Z (2015). Downregulation of survivin by shRNA inhibits invasion and enhances the radiosensitivity of laryngeal squamous cell carcinoma. Cell Biochem Biophys.

[16] Rosenberg I (2008). Radiation oncology physics: a handbook for teachers and students. Br J Cancer.

[17] Chan R, Sethi P, Jyoti A, McGarry R, Upreti M (2016). Investigating the radioresistant properties of lung cancer stem cells in the context of the tumor microenvironment. Radiat Res.

[18] Baumann M, Krause M, Hill R (2008). Exploring the role of cancer stem cells in radioresistance. Nat Rev Cancer.

[19] Gao J, Zhu Y, Nilsson M, Sundfeldt K (2014). TGF-beta isoforms induce EMT independent migration of ovarian cancer cells. Cancer Cell Int.

[20] Zhang P, Sun Y, Ma L (2015). ZEB1: at the crossroads of epithelial-mesenchymal transition, metastasis and therapy resistance. Cell Cycle.

[21] Stoddart MJ (2011). Mammalian cell viability: methods and protocols.

[22] Puck TT, Marcus PI (1956). Action of x-rays on mammalian cells. J Exp Med.

[23] Skvortsov S, Dudas J, Eichberger P, Witsch-Baumgartner M, Loeffler-Ragg J, Pritz C, Schartinger VH, Maier H, Hall J, Debbage P, Riechelmann H, Lukas P, Skvortsova I, EORTC PathoBiology Group (2014). Rac1 as a potential therapeutic target for chemo-radioresistant head and neck squamous cell carcinomas (HNSCC). Br J Cancer.

[24] de Jong MC, Ten Hoeve JJ, Grenman R, Wessels LF, Kerkhoven R, Te Riele H, van den Brekel MW, Verheij M, Begg AC (2015). Pretreatment microRNA expression impacting on epithelial-to-mesenchymal transition predicts intrinsic radiosensitivity in head and neck cancer cell lines and patients. Clin Cancer Res.

[25] Chang L, Graham PH, Hao J, Ni J, Bucci J, Cozzi PJ, Kearsley JH, Li Y (2013). Acquisition of epithelial-mesenchymal transition and cancer stem cell phenotypes is associated with activation of the PI3K/Akt/mTOR pathway in prostate cancer radioresistance. Cell Death Dis.

[26] Bergonié JT, Tribondeau L (1906). De Quelques Résultats de la Radiotherapie et Essai de Fixation d’une Technique Rationnelle. Comptes-Rendus des Séances de l’Académie des Sciences.

[27] Mendonca MS, Rodriguez A, Alpen EL (1989). Quiescence in 9L cells and correlation with radiosensitivity and PLD repair. Radiat Res.

[28] Vogin G, Foray N (2013). The law of Bergonie and Tribondeau: a nice formula for a first approximation. Int J Radiat Biol.

[29] Mazzeo E, Hehlgans S, Valentini V, Baumann M, Cordes N (2012). The impact of cell-cell contact, E-cadherin and EGF receptor on the cellular radiosensitivity of A431 cancer cells. Radiat Res.

[30] Manandhar M, Boulware KS, Wood RD (2015). The ERCC1 and ERCC4 (XPF) genes and gene products. Gene.

[31] Altieri DC (2013). Targeting survivin in cancer. Cancer Lett.

[32] Mita AC, Mita MM, Nawrocki ST, Giles FJ (2008). Survivin: key regulator of mitosis and apoptosis and novel target for cancer therapeutics. Clin Cancer Res.

[33] Altieri DC (2015). Survivin - the inconvenient IAP. Semin Cell Dev Biol.

[34] Lu B, Mu Y, Cao C, Zeng F, Schneider S, Tan J, Price J, Chen J, Freeman M, Hallahan DE (2004). Survivin as a therapeutic target for radiation sensitization in lung cancer. Cancer Res.

[35] Kappler M, Taubert H, Bartel F, Blumke K, Panian M, Schmidt H, Dunst J, Bache M (2005). Radiosensitization, after a combined treatment of survivin siRNA and irradiation, is correlated with the activation of caspases 3 and 7 in a wt-p53 sarcoma cell line, but not in a mt-p53 sarcoma cell line. Oncol Rep.

[36] Yang CT, Li JM, Weng HH, Li YC, Chen HC, Chen MF (2010). Adenovirus-mediated transfer of siRNA against survivin enhances the radiosensitivity of human non-small cell lung cancer cells. Cancer Gene Ther.

[37] Shamsabadi FT, Eidgahi MR, Mehrbod P, Daneshvar N, Allaudin ZN, Yamchi A, Shahbazi M (2016). Survivin, a promising gene for targeted cancer treatment. Asian Pac J Cancer Prev.

[38] Chen Y, Zhang F, Tsai Y, Yang X, Yang L, Duan S, Wang X, Keng P, Lee SO (2015). IL-6 signaling promotes DNA repair and prevents apoptosis in CD133+ stem-like cells of lung cancer after radiation. Radiat Oncol.

[39] Orimo A, Gupta PB, Sgroi DC, Arenzana-Seisdedos F, Delaunay T, Naeem R, Carey VJ, Richardson AL, Weinberg RA (2005). Stromal fibroblasts present in invasive human breast carcinomas promote tumor growth and angiogenesis through elevated SDF-1/CXCL12 secretion. Cell.

[40] Berdiel-Acer M, Bohem ME, Lopez-Doriga A, Vidal A, Salazar R, Martinez-Iniesta M, Santos C, Sanjuan X, Villanueva A, Molleví DG (2011). Hepatic carcinoma-associated fibroblasts promote an adaptative response in colorectal cancer cells that inhibit proliferation and apoptosis: nonresistant cells die by nonapoptotic cell death. Neoplasia.

[41] Goncalves-Ribeiro S, Guillen Diaz-Maroto N, Berdiel-Acer M, Soriano A, Guardiola J, Martinez-Villacampa M, Salazar R, Capellà G, Villanueva A, Martínez-Balibrea E, Molleví DG (2016). Carcinoma-associated fibroblasts affect sensitivity to oxaliplatin and 5FU in colorectal cancer cells. Oncotarget.

[42] Dudas J, Fullar A, Bitsche M, Schartinger V, Kovalszky I, Sprinzl GM, Riechelmann H (2011). Tumor-produced, active interleukin-1beta regulates gene expression in carcinoma-associated fibroblasts. Exp Cell Res.

[43] Dudas J, Fullar A, Romani A, Pritz C, Kovalszky I, Hans Schartinger V, Sprinzl GM, Riechelmanna H (2013). Curcumin targets fibroblast-tumor cell interactions in oral squamous cell carcinoma. Exp Cell Res.

[44] Hassona Y, Cirillo N, Heesom K, Parkinson EK, Prime SS (2014). Senescent cancer-associated fibroblasts secrete active MMP-2 that promotes keratinocyte dis-cohesion and invasion. Br J Cancer.

[45] Liu Y, Zhang M, Qian J, Bao M, Meng X, Zhang S, Zhang L, Zhao R, Li S, Cao Q, Li P, Ju X (2015). miR-134 functions as a tumor suppressor in cell proliferation and epithelial-to-mesenchymal transition by targeting KRAS in renal cell carcinoma cells. DNA Cell Biol.

[46] Friedrich K, Dolznig H, Han X, Moriggl R (2017). Steering of carcinoma progression by the YIN/YANG interaction of STAT1/STAT3. Biosci Trends.

[47] Korkaya H, Kim GI, Davis A, Malik F, Henry NL, Ithimakin S, Quraishi AA, Tawakkol N, D'Angelo R, Paulson AK, Chung S, Luther T, Paholak HJ (2012). Activation of an IL6 inflammatory loop mediates trastuzumab resistance in HER2+ breast cancer by expanding the cancer stem cell population. Mol Cell.

[48] Milagre CS, Gopinathan G, Everitt G, Thompson RG, Kulbe H, Zhong H, Hollingsworth RE, Grose R, Bowtell DD, Hochhauser D, Balkwill FR (2015). Adaptive upregulation of EGFR limits attenuation of tumor growth by neutralizing IL6 antibodies, with implications for combined therapy in ovarian cancer. Cancer Res.

[49] Olsen J, Kirkeby LT, Olsen J, Eiholm S, Jess P, Gogenur I, Troelsen JT (2015). High interleukin-6 mRNA expression is a predictor of relapse in colon cancer. Anticancer Res.

[50] Docheva D, Padula D, Popov C, Weishaupt P, Pragert M, Miosge N, Hickel R, Böcker W, Clausen-Schaumann H, Schieker M (2010). Establishment of immortalized periodontal ligament progenitor cell line and its behavioural analysis on smooth and rough titanium surface. Eur Cells Mater.

[51] Valente V, Teixeira SA, Neder L, Okamoto OK, Oba-Shinjo SM, Marie SK, Scrideli CA, Paçó-Larson ML, Carlotti CG (2014). Selection of suitable housekeeping genes for expression analysis in glioblastoma using quantitative RT-PCR. Ann Neurosci.

[52] Skvortsova I, Skvortsov S, Stasyk T, Raju U, Popper BA, Schiestl B, von Guggenberg E, Neher A, Bonn GK, Huber LA, Lukas P (2008). Intracellular signaling pathways regulating radioresistance of human prostate carcinoma cells. Proteomics.

[53] Schartinger VH, Galvan O, Riechelmann H, Dudas J (2012). Differential responses of fibroblasts, non-neoplastic epithelial cells, and oral carcinoma cells to low-level laser therapy. Support Care Cancer.

[54] Pike MC, Alper T (1964). A method for determining dose-modification factors. Br J Radiol.

