# Correction: Epithelial-mesenchymal crosstalk induces radioresistance in HNSCC cells

**DOI:** 10.18632/oncotarget.27254

**Published:** 2019-10-08

**Authors:** Teresa Bernadette Steinbichler, Alshaimaa Abdelmoez, Veronika Maria Metzler, Daniel Dejaco, Herbert Riechelmann, Jozef Dudas, Ira-Ida Skvortsova

**Affiliations:** ^1^ Department of Otorhinolaryngology, Medical University of Innsbruck, Innsbruck, Austria; ^2^ Department of Therapeutic Radiology and Oncology, Medical University of Innsbruck, Innsbruck, Austria

**This article has been corrected:** The author names below were unintentionally reversed in the listing. The correct author names are as follows:

Alshaimaa Abdelmoez

Veronika Maria Metzler

Daniel Dejaco

Herbert Riechelmann

Jozef Dudas

Ira-Ida Skvortsova

Original article: Oncotarget. 2018; 9:3641–3652. 3641-3652. https://doi.org/10.18632/oncotarget.23248

